# Super‐Refractory Status Epilepticus ( **SRSE** ) in a Patient With Compound Heterozygous *OPA1* Variants: Case Report and Literature Review

**DOI:** 10.1002/acn3.70287

**Published:** 2026-02-11

**Authors:** Pouria Mohammadi, Lara Basovic, Christopher Michael McGraw

**Affiliations:** ^1^ Myelin Disorders Clinic, Children's Medical Centre, Paediatric Centre of Excellence Tehran University of Medical Sciences Tehran Iran; ^2^ Northwestern University, Feinberg School of Medicine Chicago Illinois USA; ^3^ Massachusetts General Hospital, Harvard Medical School Boston Massachusetts USA

**Keywords:** mitochondrial disease, mitochondrial dynamics, OPA1, SRSE, super‐refractory status epilepticus

## Abstract

**Objective:**

Super‐Refractory Status Epilepticus (SRSE) is a rare, life‐threatening neurological emergency with unclear etiology in many cases. Mitochondrial dysfunction, often due to disease‐causing genetic variants, is increasingly recognized as a cause, with each gene producing distinct pathophysiological mechanisms.

**Methods:**

We describe the detailed clinical, neurophysiological, neuroimaging, and molecular findings of a 19‐year‐old female with SRSE associated with compound heterozygous variants in *OPA1*, a key gene for mitochondrial inner membrane fusion and cristae maintenance. In addition, a literature review was performed, identifying 16 previously published cases reporting one or both of the variants observed in the present case.

**Results:**

Despite a longstanding history of generalized hypotonia, celiac disease, optic atrophy, cerebellar ataxia, and progressive motor decline, the proband had no prior history of seizures. She developed super‐refractory status epilepticus with occipital‐predominant epileptiform activity and MRI showing transient diffusion restriction in the right parieto‐occipital cortex and cerebellum. Genetic testing revealed a frameshift variant (p.Val903GlyfsTer3) and a missense variant (p.Ile382Met) in the GTPase domain, known to impair mitochondrial fusion. Unlike *POLG* or MELAS‐associated seizures, typically driven by severe mtDNA depletion and respiratory chain failure, *OPA1* dysfunction usually spares mtDNA copy number but disrupts mitochondrial dynamics. In severe biallelic loss‐of‐function, a “second‐hit” stressor may trigger a diffuse energy crisis and catastrophic seizures.

**Interpretation:**

This case of mitochondrial SRSE in a patient with no known infectious, autoimmune, or structural cause emphasizes the possible role of genetic background and mitochondrial disorders in the development of the disease. This case highlights a rare mitochondrial subtype of RSE, emphasizing the need to consider energy metabolism defects in unexplained refractory status epilepticus.

## 1 Introduction

Super‐Refractory Status Epilepticus (SRSE) with unclear etiology is one of the most challenging neurologic emergencies in clinical practice. It refers to a clinical presentation where a young person without a history of seizures suddenly develops status epilepticus that does not respond to standard treatments [1]. When there is no identifiable trigger, such as infection, trauma, or structural brain abnormality, clinicians suspect that an autoimmune or parainfectious process may be at play, and they initiate broad‐spectrum immunosuppressive treatments [2, 3]. In certain cases, it proves to be lifesaving; however, in others, when it turns out that autoimmune disease was not involved at all, these same treatments can be harmful, for example, causing severe infections or multiorgan failure [2]. Despite extensive advances in imaging, infectious, and autoimmune investigations, the etiology of SRSE remains unclear in many patients [4]. Recent studies suggest that some of these cases may have underlying genetic or mitochondrial disorders, with status epilepticus as the first clinical manifestation [5]. Due to the diagnostic uncertainty in many SRSE cases, genetic testing has gradually emerged as a promising tool for identifying the underlying causes of the disease.

Recent findings indicate that mitochondrial and synaptic dysfunctions, which in many cases are caused by genetic variants, can play an important role in the development and progression of SRSE disease. In this regard, several disease‐causing variants in genes including *POLG* [6], *DNM1L* [7], *OPA1* [8], *TBC1D24* [9], *FASTKD2* [10], *PIGG* (Kellogg et al. [11]), and *FADD* [12] have been reported in patients with refractory status epilepticus (RSE) or severe epileptic encephalopathies, which could indicate a potential role of these genes in the pathophysiology of these disorders. These genes are involved in important cellular functions, including mitochondrial DNA replication, mitochondrial fission and fusion processes, synaptic vesicle transport, and apoptosis. Despite these associations, the contribution of these genes to the onset and severity of RSE and systematic genotype–phenotype correlations is still not well understood.

The *OPA1* gene encodes a dynamin‐related GTPase that is associated with the inner mitochondrial membrane and plays a key role in regulating mitochondrial fusion, cristae architecture, mitochondrial DNA (mtDNA) maintenance, and apoptosis [13, 14]. Heterozygous disease‐causing variants in *OPA1* typically cause dominant optic atrophy (DOA) [15], and homozygous variants can cause a broader and more severe spectrum of clinical syndromes, particularly DOA‐plus and Behr syndrome [16]. Beyond visual impairment, clinical manifestations of *OPA1* variants can include hypotonia, ataxia, sensorineural hearing loss, neuropathy, and progressive encephalopathy. This phenotypic diversity highlights the critical role of *OPA1* in mitochondrial function [16, 17].

Here, we describe a 19‐year‐old female with mitochondrial associated‐SRSE caused by compound heterozygous variants in the *OPA1* gene. Despite a longstanding history of generalized hypotonia, celiac disease, legal blindness due to optic atrophy, cerebellar ataxia, and progressive motor decline, she had no prior history of seizures. While this case does not meet the full criteria for New‐Onset Refractory Status Epilepticus (NORSE), it was managed similarly due to the lack of prior epilepsy and the unclear association of the *OPA1* gene with epilepsy or RSE. This case highlights the potential role of genetic background and mitochondrial disorders in the development of the disease and emphasizes the need to consider energy metabolism defects in unexplained SRSE.

## 2 Materials and Methods

### 2.1 Ethical Compliance

Legal guardians provided written informed consent for participation and publication of the photographs. This study was conducted in accordance with the principles of the Declaration of Helsinki.

### 2.2 Clinical Evaluation and In Silico Analysis

Genetic counseling was initially provided to a 19‐year‐old female patient with a known mitochondrial disorder who presented with SRSE. A detailed clinical evaluation was conducted through retrospective review of her medical history, including genetics and neurology follow‐ups, inpatient notes during the SRSE episode, imaging findings, EEG data, and results from molecular diagnostics. The aim was to characterize the full phenotypic presentation and assess the possible link between her genetic background and the development of SRSE. To visualize the structural and functional context of the identified *OPA1* variants, several in silico tools were applied. The domain organization of OPA1 was annotated based on UniProt (UniProtKB: O60313). A three‐dimensional structural model was generated and visualized using PyMOL software. Evolutionary conservation of the affected residues was evaluated with the ConSurf server. Variant intolerance across the OPA1 protein was assessed using MetaDome, which integrates population‐based variation tolerance scores.

### 2.3 Literature Review

A comprehensive literature review was performed using PubMed and Web of Science databases up to June 2025. The search strategy included keywords such as “*OPA1* [title/abstract] variant,” “Behr syndrome [title/abstract],” “Mitochondrial SRSE [title/abstract],” and “Refractory Status Epilepticus [title/abstract].” All published case reports and clinical studies involving patients with biallelic or compound heterozygous *OPA1* variants were screened for inclusion. Relevant data were systematically extracted, including patient demographics, genotype and zygosity, clinical manifestations (especially seizures and neurological findings), imaging results, EEG patterns, and outcomes. Special attention was given to reports documenting refractory status epilepticus or related mitochondrial phenotypes associated with *OPA1* variants (Table 1).

**TABLE 1:** Clinical features and genotypes of cases with (c.1146A>G) and/or (c.2708_2711del) *OPA1* variants.

Case	Gender/Age	Age at onset of first symptoms	c.DNA/Amino acid change*OPA1* gene	Zygosity	Disease/Inheritance	Neurologic features	Optic atrophy	Seizure/Epilepsy	MRI	Other features	EEG	Study (PMID)
1	F/19 years	Infancy	NM_015560.3: c.2708_2711del; (p.Val903GlyfsTer3)/ NM_015560.3:c.1146A>G; (p.Ile382Met)	Compound Het	OPA1‐related mitochondrial Super‐refractory status epilepticus/AR	Hypotonia, Ataxia, Dysarthria, Polyneuropathy, Spasticity, Developmental delay, Muscle wasting, Wheelchair‐bound since 12	Yes (legal blindness)	Super‐refractory status epilepticus at 19 years	Transient diffusion restriction in right parieto‐occipital cortex and cerebellum, Hypermetabolism	Anxiety, Celiac disease, Syndactyly, clinodactyly, Failure to thrive	Persistent right‐posterior LPDs with superimposed rhythmic/fast activity (LPDs+R/F), interictal–ictal continuum, disorganized background, no sleep architecture	Present study
2	M/8 years F/3 years (two siblings)	Infancy	NM_015560.3: c.2708_2711del ; (p.Val903GlyfsTer3)/ NM_015560.3:c.1146A>G; (p.Ile382Met)	Compound Het	OPA1‐related disorder/AR	Sensory ataxia and peripheral neuropathy, Hypotonia, Nystagmus	Yes	No	Mild periventricular leukomalacia	Gastrointestinal dysmotility, dysphagia, constipation, Legally blind	NA	Schaaf et al. [18]
3	F/12 years	Infancy	NM_015560.3; c.1963_1964dupAT; (p.Lys656Serfster16)/ c.1146A>G; (p.Ile382Met)	Compound Het	OPA1‐related complex early onset neurological disorder/AR	Metabolic stroke, Global developmental delay, Nystagmus, Progressive ataxia, Dysarthria, Tremor, Motor and language delay, Learning impairment, Pyramidal signs	Yes	Prolonged febrile seizure, Focal seizure clusters, Myoclonic jerks	Mild central and deep white matter multifocal T2 hyperintensities, Right parietal, acute infarct with restricted diffusion and edema, Parenchymal loss in in both parietal lobes	Bilateral foot drop	Background slowing in parieto‐occipital areas; Intermittent spikes	Zerem et al. [19]
4	M/8 years (deceased)	Infancy	NM_015560.3; c.1146A>G; (p.Ile382Met)/ c.190_194del; (p.Ser64AspfsTer7)	Compound Het	OPA1‐related mitochondrial disorder/AR	Nystagmus, Psychomotor delay, Hypotonia, Hemiparesis, Progressive ataxia, Severe peripheral neuropathy, Mental retardation	Yes	Myoclonic epileptic seizures (age 4), controlled initially	Putaminal necrosis, cerebellar atrophy, mesencephalon/pons swelling, thin corpus callosum, cortical changes.	Elevated lactate, Hepatopathy, Vomiting, Growth delay, Multiorgan failure	Poorly organized activity; sharp waves in occipital regions; later theta‐delta asymmetry (right hemisphere depression)	Nasca et al. [13]
5	F/14	4–6 years	NM_015560.3: c.1146A>G; (p.Ile382Met)/ NM_130837.3: c.2962G>T; (p.Val988Phe)	Compound Het	OPA1‐ related disorder/AR	Mild ptosis, Strabismus, Axonal sensory neuropathy, Dysdiadochokinesia, Dysmetria, Mild ataxia, Areflexia	Yes	No	Optic nerves and chiasm atrophy	Pes cavus, Normal cognitive; Normal psychomotor development	Normal	Nasca et al. [13]
6	F/50 years	2 years	NM_015560.3:c.1146A>G; (p.Ile382Met)/ NM_130837.3:c.610+364G>A	Compound Het	OPA1‐related disorder/AR	Cerebellar ataxia, Ophthalmoplegia, Spasticity, Muscle atrophy, Peripheral neuropathy, Bilateral ptosis	Yes	No	Cerebellar atrophy, Atrophy of the upper cervical spinal cord	High SARA score (34/40), CK & lactate normal	NA	Bonifert et al. [20]
7	M/48 years	2 years	NM_015560.3:c.1146A>G; (p.Ile382Met)/ NM_130837.3:c.610+364G>A	Compound Het	OPA1‐related disorder/AR	Cerebellar ataxia, Ophthalmoplegia, Spasticity, Muscle atrophy, Bilateral ptosis	Yes	No	Cerebellar atrophy, Atrophy of the upper cervical spinal cord	High SARA score (30/40), CK & lactate normal	NA	
8	M/46 years	2 years	NM_015560.3:c.1146A>G; (p.Ile382Met)/ NM_130837.3:c.610+364G>A	Compound Het	OPA1‐related disorder/AR	Cerebellar ataxia, Ophthalmoplegia, Spasticity, Muscle atrophy, Bilateral ptosis	Yes	No	Cerebellar atrophy, Atrophy of the upper cervical spinal cord	High SARA score (29/40), CK & lactate normal	NA	
9	F/13 years	1 year	NM_015560.3:c.1146A>G; (p.Ile382Met)/ NM_130837.3: c.1316_1317insA; (p.Asn440LysfsTer14)	Compound Het	OPA1‐related disorder/AR	Sensory predominant peripheral neuropathy and truncal as well as limb ataxia, Muscle atrophy	Yes	No	NA	Normal CK & lactate	NA	
10	M/20 years	At birth	NM_015560.3:c.1146A>G; (p.Ile382Met)/ NM_015560.3: c.1705+1G>T	Compound Het	Behr syndrome/AR	Spastic paraparesis, cerebellar ataxia, Sensory ataxia, Peripheral neuropathy	Yes	No	Mild cerebellar atrophy; CSF lactate accumulation	3‐methylglutaconic aciduria, wheelchair‐bound, bilateral talipes equinovarus	NA	Carelli et al. [21]
11	M/17 months	At birth	NM_130837.3: c.1311A>G (p.lIle437Met)/ NM_130837.3: c.2287del (p.Ser763ValfsTer15)	Compound Het	Behr syndrome/AR	Developmental delay, Limb and gait ataxia, Hypotonia, Hypoactive reflexes, Nystagmus	Yes	Status epilepticus and intractable seizures	Multiple metabolic strokes in his left thalamus, left occipital lobe, and left frontal lobe	Low myopia an astigmatism. No signs of peripheral neuropathy Grossly normal strength, sensation and muscle tone, Normal hearing	Abnormal	Othman et al. [22]
12	M/14 years	18 months	NM_015560.3:c.1146A>G; (p.Ile382Met)/ NM_015560.3:c.2470C>T (p.Arg824Ter)	Compound Het	Early‐onset Behr syndrome/AR	Ataxia, Dysmetria, Rigidity, Sensory neuropathy, Progressive motor decline	Yes	No	Mild atrophy of the cerebellar vermis and involvement of the white matter, with mild periventricular T1 hypersignals and a focal lesion at the posterior end of the lenticular nucleus	Deafness	NA	Bonneau et al. [23]
13	F/4 years	14 months	NM_015560.3:c.1146A>G; (p.Ile382Met)/ NM_015560.3:c.1669C>T (p.Arg557Ter)	Compound Het	Early‐onset Behr syndrome/AR	Mild ataxia, Slight tremor, Axonal sensory neuropathy	Yes	No	Normal at age 4	Constipation, visual disability	NA	Bonneau et al. [23]
14	M/15 years	36 months	NM_015560.3:c.1146A>G; (p.Ile382Met)/ NM_015560.3: c.1459G>A (p.Glu487Lys)	Compound Het	Early‐onset Behr syndrome/AR	Ataxia, Peripheral neuropathy, Vermian Atrophy	Yes	No	Optic chiasm hypoplasia, cerebellar vermian atrophy	Legal blindness, frequent falls	NA	Bonneau et al. [23]
15	F/11 years	12 months	NM_015560.3:c.2708_2711del p.(Val903GlyfsTer3)/NM_015560.3: c.1204G>A; (p.Val402Met)	Compound Het	Early‐onset Behr syndrome/AR	Trunk ataxia, Sensorimotor neuropathy, Cerebellar ataxia, Dysarthria	Yes	No	Cerebellar vermis atrophy, lactate peak	Recurrent vomiting, mild dysarthria	NA	Bonneau et al. [23]
16	M/7 years	At birth	NM_015560.3:c.2287del; (p.Ile763LeufsTer37)/NM_130837.3: c.1311A>G (p.lIle437Met)	Compound Het	Behr syndrome/AR	Generalized hyporeflexia, Intention tremors, Ataxic gait, Developmental delay, Nystagmus	Yes	Super‐refractory status epilepticus	Normal at 17 m, later multiple non‐vascular diffusion restrictions (left thalamus, parietal, occipital, frontal), Metabolic stroke	Feeding difficulties, aspiration, gastrostomy, sensorimotor regression, partial recovery with rehab	Multifocal epileptiform discharges, left parasagittal SE, right temporal NCSE, posterior polyspike discharges	Jagadish et al. [8]
17	F/28 years	Childhood	NM_015560.3:c.2708_2711del p.(Val903GlyfsTer3)	Het	Dominant Optic Atrophy plus/AD	Spastic paraplegia, Brisk reflexes, Ankle clonus, Mild muscle atrophy	Yes	No	Normal	Duane Retraction Syndrome (left eye), migraine with aura, patent foramen ovale	NA	Pretegiani et al. [24]

Abbreviations: AD, autosomal dominant; AR, autosomal recessive; Compound Het, compound heterozygous; F, female; Het, heterozygous; Hom, homozygous; M, male; N.A, not available.

## 3 Result

### 3.1 Case History

The proband is a 19‐year‐old female diagnosed with a clinical presentation of mitochondrial disorder, which was consistent with the identified biallelic variants in *OPA1*. Primary clinical presentations included nystagmus from infancy, hypotonia, celiac disease, progressive optic atrophy leading to legal blindness, sensory polyneuropathy, gait abnormalities, cerebellar ataxia, developmental delays, and syndromic findings such as 2–3 toe syndactyly and fifth finger clinodactyly. She has been in a wheelchair since the age of 12, which has been accompanied by fatigue, muscle cramps, and spasticity. She was completely dependent for all activities of daily living and had long‐term urinary incontinence and chronic malnutrition. She had no history of seizures or epilepsy.

### 3.2 Molecular Genetics Findings

**FIGURE 1:**
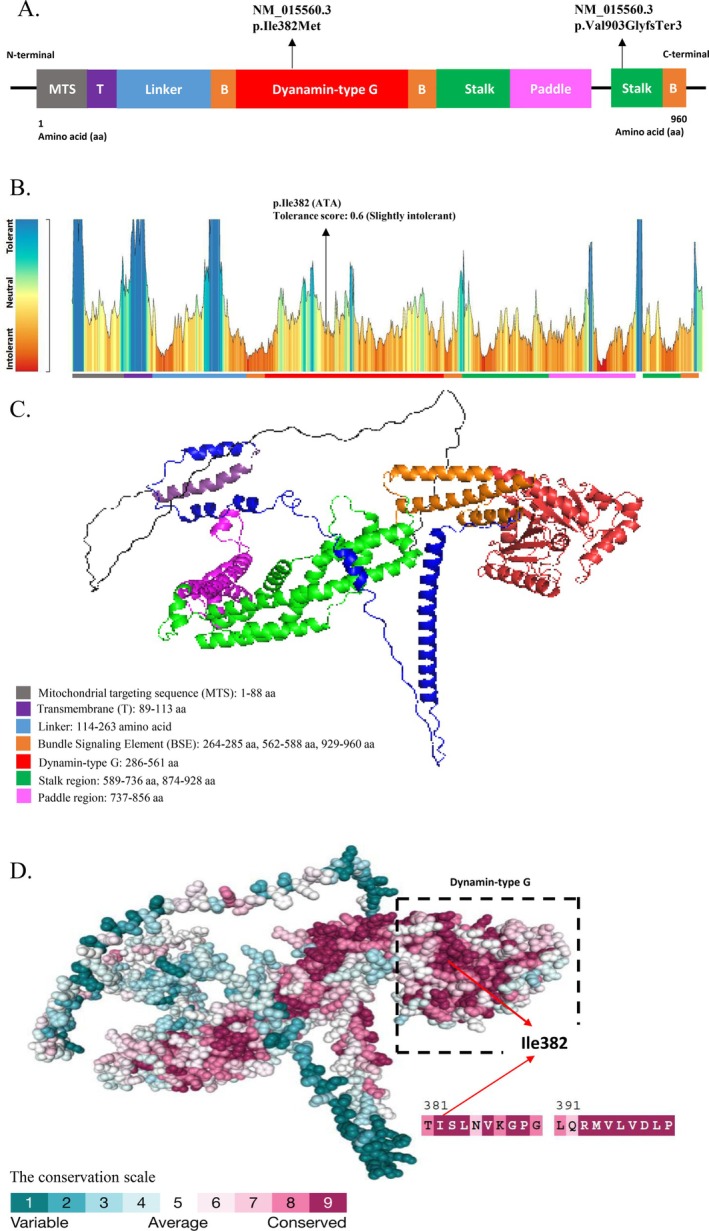
Domain architecture and structural mapping of *OPA1* variants. (A) Linear schematic representation of the OPA1 protein (960 aa) showing its functional domains: Mitochondrial targeting sequence (MTS), transmembrane (T) region, linker, bundle signaling element (BSE), dynamin‐type G domain, stalk regions, and paddle region. The identified variants p.Ile382Met and p.Val903GlyfsTer3 are mapped to their respective domains. (B) Missense tolerance landscape generated by MetaDome server, indicating the relative tolerance/intolerance of each amino acid position. The p.Ile382Met variant lies in a slightly intolerant region (tolerance score = 0.6). (C) Three‐dimensional structural model of OPA1 generated and visualized with PyMOL software, color‐coded according to domains and annotated with the positions of key regions. (D) Amino acid conservation analysis performed using ConSurf server, mapping evolutionary conservation scores onto the protein surface. The Ile382 residue, located within the dynamin‐type G domain, is highly conserved, suggesting functional importance.

In the proband, a compound heterozygous variant was identified in *OPA1* (OMIM * 605290). The first variant was a maternally inherited frameshift deletion [chr3(hg38)‐193667167‐CAGTT>C NM_015560.3:c.2708_2711del; p.Val903GlyfsTer3, exon 27 of 29]. This variant creates a shift in the reading frame starting at codon 903. The new reading frame ends in a stop codon 3 positions downstream. This variant is classified as pathogenic according to ACMG (class1; PVS1, PM2, PP5, PP4) and ClinVar. It was also reported as disease causing by HGMD. The second variant was a paternally inherited missense variant [chr3(hg38)‐193642998‐A>G NM_015560.3:c.1146A>G; p.Ile382Met; exon 12 of 29], also referenced as NM_130837.3: c.1311A>G, located in the GTPase domain. This variant causes an amino acid change from Ile to Met at position 382. This variant is classified as pathogenic according to ACMG (class1; PP3, PP1, PM1, PM2, PP5, PS4, PP4) and Conflicting Interpretations Of Pathogenicity (8 pathogenic, 3 likely pathogenic, 11 VUS submissions) based on ClinVar. It was also reported as disease causing by HGMD. The domain architecture and structural mapping of these variants are shown in Figure 1. The *OPA1* gene encodes a Mitochondrial Dynamin‐related GTPase (UniProtKB: O60313) protein that localizes to the inner mitochondrial membrane and regulates several important cellular processes including the stability of the mitochondrial network, mitochondrial bioenergetic output, and sequestration of proapoptotic cytochrome C oxidase molecules within the mitochondrial cristae spaces [16]. The structure of the OPA1 protein includes: Mitochondrial targeting sequence (MTS) at the N‐terminus, cleaved after import, Transmembrane domain (TM) anchoring it to the inner membrane, GTPase domain, a conserved region responsible for GTP hydrolysis, Bundle signaling element (BSE) connecting GTPase to effector regions, GTPase effector domain (GED) involved in oligomerization and activity regulation, and Stalk domain, aiding membrane interaction [25, 26].

Disease‐causing variants in *OPA1* are associated with autosomal recessive Mitochondrial DNA depletion syndrome 14 (encephalocardiomyopathic type) (OMIM:616896), autosomal recessive Behr syndrome (OMIM:210000), autosomal dominant Optic atrophy (OMIM:165500), and autosomal dominant Optic atrophy plus syndrome (OMIM:125250).

### 3.3 Patient's Presentation With SRSE

**FIGURE 2:**
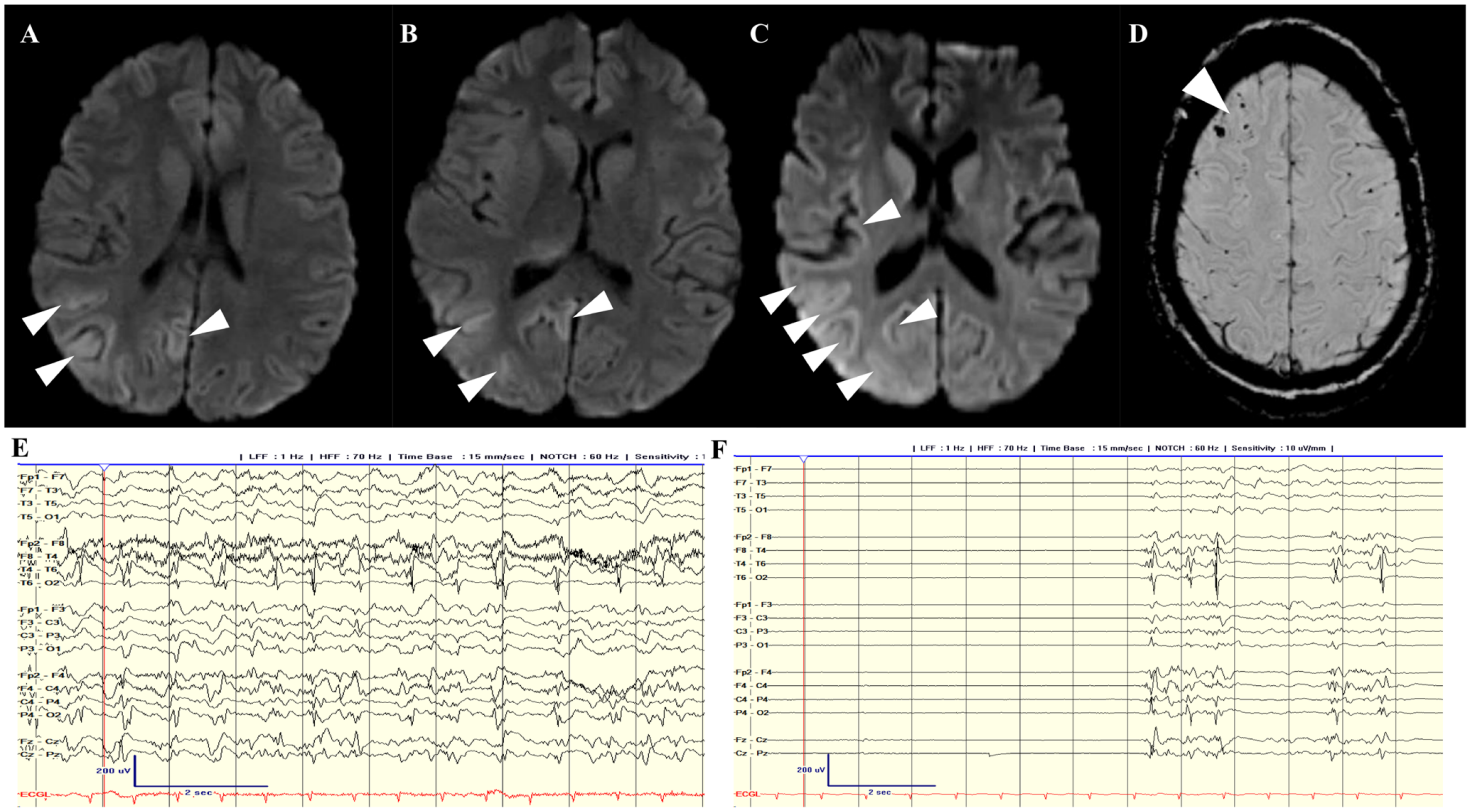
(A–C) Diffusion‐weighted imaging (DWI) brain MRI from early (A, B), obtained 4 days after the onset of seizures, and later (C), was taken 40 days after the onset, in patient's course demonstrating persistent diffusion restriction involving right parietooccipital areas (arrowheads). (D) Susceptibility weighted imaging (SWI) brain MRI showing right frontal microbleed (arrowhead), obtained 42 days after seizure onset. (E, F) Electroencephalography (EEG) demonstrating abundant right posterior epileptiform discharges and LPDs prior to propofol (E) and during burst suppression (F).

At age 19, the proband experienced her first generalized tonic–clonic seizure, which was preceded by headache, fatigue, and vomiting. Subsequently, she developed focal status epilepticus, characterized by leftward gaze deviation, head turning, and rhythmic movements of the left upper limb. Initial electroencephalogram (EEG) revealed right posterior epileptiform discharges and serial MRI showed transient diffusion restriction in the right parieto‐occipital cortex and cerebellum consistent with seizure‐related edema (Figure 2). Due to clinical deterioration, she was intubated and admitted to the intensive care unit at Maine Medical Center (MMC). Despite initiation of treatment with midazolam, ketamine, and pentobarbital to induce burst suppression, seizures remained refractory.

Following a brief clinical improvement, she had experienced recurrent seizures and was re‐initiated on continuous phenobarbital therapy. Her condition was classified as super‐refractory status epilepticus (SRSE), and she was transferred to Massachusetts General Hospital (MGH) for further care.

At MGH, continuous EEG revealed frequent right posterior 1–2 Hz lateralized periodic discharges (LPDs) with superimposed rhythmic and fast activity (LPDs+*R*/F), consistent with the interictal–ictal continuum. Burst suppression was intermittently achieved with pentobarbital, with suppression ratios exceeding 90% following boluses, although maintaining this level proved difficult beyond a few hours. Earlier attempts at burst suppression using midazolam were paradoxically associated with increased epileptiform activity, suggesting benzodiazepine refractoriness. The EEG demonstrated a persistently disorganized background, without identifiable features of normal sleep architecture. MRI and FDG‐PET showed persistent right parieto‐occipital abnormalities and hypermetabolism, indicating an active seizure focus and mitochondrial dysfunction. CSF analyses, including autoimmune and infectious panels, were non‐inflammatory and unremarkable. A repeat lumbar puncture later in the course similarly showed normal cell counts and protein, and cultures remained negative.

Multidrug therapy including midazolam, pentobarbital, propofol, levetiracetam, lacosamide, phenytoin, perampanel, and clobazam resulted in limited clinical improvement. Due to the patient's known genetic diagnosis with negative inflammatory work‐up, empiric immunosuppression was not pursued. Then, ganaxolone was started and a ketogenic diet was initiated. Over the next week, the EEG gradually improved and transformed into generalized rhythmic delta activity (GRDA) without seizures. The recurrence of seizures was temporally associated with a missed dose of perampanel and a concurrent urinary tract infection, suggesting a potential acute trigger. Her course was further complicated by aspiration pneumonia, recurrent UTIs, transaminitis, and a right frontal microbleed. She underwent temporary tracheostomy and G‐tube placement, both of which were successfully stabilized or reversed before discharge. Of note, the patient's clinical course was not complicated by any known adverse effects of propofol. In particular, there were no features suggestive of propofol infusion syndrome (PRIS), which is characterized by metabolic acidosis (base deficit > 10 mmol/L), arrhythmia, heart failure, renal insufficiency, hepatomegaly, and/or rhabdomyolysis. None of these abnormalities were observed during the patient's hospitalization.

After prolonged ICU care and multidisciplinary rehabilitation, the patient was extubated, alert, and intermittently verbal, and her cognitive and neurological recovery was slow but steady, although still below her pre‐illness baseline. At discharge, she was maintained on six antiseizure medications (levetiracetam, lacosamide, phenobarbital, phenytoin, perampanel, and ganaxolone) along with the ketogenic diet. A gradual weaning plan was recommended, beginning with ganaxolone.

## 4 Discussion

### 4.1 Molecular and Functional Basis of *OPA1* Variants

In this study, we describe a 19‐year‐old female with compound heterozygous variants in the *OPA1* gene presenting with Mitochondrial SRSE, which is a specific subtype of status epilepticus that may result from primary mitochondrial dysfunction, particularly due to disease‐causing variants in genes responsible for oxidative phosphorylation, mitochondrial dynamics, RNA processing, or metabolic homeostasis [10, 27]. Unlike autoimmune or cryptogenic forms of NORSE, in which immune dysregulation or unknown inflammatory processes are often suspected as the underlying cause, mitochondrial SRSE is postulated to arise due to primary failure in cellular energy production. Dysfunction of the mitochondrial respiratory chain impairs ATP production and increases reactive oxygen species, which can cause neuronal damage and seizure activity [28, 29, 30].

This patient was initially managed as NORSE, due to the unclear association with her neurological condition and biallelic *OPA1* variants at presentation, but we acknowledge that SRSE is the more accurate term for her presentation. According to current consensus, NORSE requires new‐onset RSE in a patient without active epilepsy or another relevant neurologic disorder and without a clear acute/active structural, toxic, or metabolic cause [4, 31]. However, this definition is not always consistently applied in the literature. For example, the authors Hanin et al. [32] included patients with autoimmune encephalitis and prior epilepsy (but no prior RSE) in their group of NORSE patients, even though technically deterioration of an existing cause of epilepsy is not meant to be included in NORSE; meanwhile, the authors did exclude cases of RSE due to epilepsy from a non‐autoimmune cause. As clinicians, we contend that our patient's presentation with RSE was certainly less well explained by her prior neurologic diagnoses than that of RSE in a patient with prior autoimmune epilepsy, but only the latter is considered NORSE. These observations highlight semantic distinctions in defining NORSE versus non‐NORSE RSE (of new onset) that may be relevant to patients with pre‐existing mitochondrial or other non‐inflammatory etiologies of RSE.

Our patient harbors two variants in *OPA1*. The first variant (c.2708_2711del; p.Val903GlyfsTer3) leads to the activation of the nonsense‐mediated mRNA decay (NMD) mechanism. Quantitative transcript analysis, performed in RNA isolated from peripheral blood samples and in some cases from lymphoblastoid cell lines, demonstrated that the mutant allele contributes only 23%–34% of total *OPA1* mRNA, consistent with degradation via NMD [33]. The second variant (c.1146A>G; p. Ile382Met) causes a structurally impaired OPA1 protein that disrupts mitochondrial fusion, GTPase folding, and cristae organization [20, 34]. It reduces mitochondrial fusion by ~40% and destabilizes mtDNA in yeast [35], impairs mitophagy in compound genotypes [36], and induces mitochondrial fragmentation and energy failure under stress in mammalian cells [17, 37]. iPSC‐based modeling further supports its pathogenic mechanism [38].

The combination of a null allele variant and a hypomorphic variant in *OPA1* in this patient leads to a decrease in OPA1 protein function, disrupting mitochondrial network dynamics and increasing neuronal vulnerability to metabolic stress [20, 34]. The clinical manifestations in this patient are consistent with cellular and structural models of *OPA1* dysfunction, suggesting that these genetic variants may confer a high risk of neuronal vulnerability. This case supports a direct link between impaired *OPA1* function and the development of SRSE, even in the absence of infectious, autoimmune, or structural causes.

In addition to functional evidence, several clinical reports have emphasized the epileptogenic potential of the p.Ile382Met variant, particularly when inherited in trans with a truncating allele. In this regard, Jagadish et al. [8] reported a child with compound heterozygous *OPA1* variants (p.Ile382Met and c.2287del). At the age of 7 years, he presented with recurrent episodes of super‐refractory status epilepticus and metabolic stroke due to underlying mitochondrial dysfunction associated with *OPA1* dysfunction. Similarly, Al Othman et al. [22] reported exactly the variants of the Jagadish et al. [8] study in a patient with status epilepticus and multifocal metabolic strokes [22]. By contrast, cases with compound heterozygosity for two missense variants such as p.Ile382Met with p.Val988Phe or p.Ile382Met with p.Glu487Lys, reported by Nasca et al. [13] and Bonneau et al. [23], respectively, showed later‐onset optic atrophy and multisystem involvement but no seizures, epilepsy, or NORSE, supporting the notion that missense + truncating combinations confer a higher epileptogenic risk than missense + missense genotypes.

However, the phenotypic spectrum of *OPA1*‐related disorders can be highly variable, even among patients with similar genotypes. Notably, Schaaf et al. [18] reported two siblings with the same compound heterozygous variants (c.2708_2711delTTAG, c.1146A>G) in the *OPA1* who exhibited severe ataxia, hypotonia, gastrointestinal dysmotility, dysphagia, and severe, early‐onset optic atrophy without epilepsy (Given the limited follow‐up duration in that study, it remains possible that seizures might have developed later). This suggests that additional modifying factors such as genetic background (modifier genes particularly those affecting mitochondrial dynamics, antioxidant capacity, or excitability thresholds) and epigenetic regulation (differential methylation or allelic expression bias) or environmental factors may influence the clinical trajectory towards SRSE rather than a non‐epileptic phenotype.

### 4.2 Mechanisms and Triggers of Seizure Vulnerability

The development of mitochondrial SRSE in our patient represents a rare and severe manifestation within the phenotypic spectrum associated with biallelic *OPA1* variants. This clinical course is consistent with a “threshold model” of mitochondrial failure, in which subclinical dysfunction remains tolerated under basal conditions but rapidly collapses under physiological stress [28, 39].

Importantly, serial brain MRI in our patient revealed transient diffusion restriction in the right parieto‐occipital cortex and cerebellum, with corresponding right‐posterior epileptiform discharges on EEG, and FDG‐PET hypermetabolism in the same region. These findings suggest that an acute metabolic crisis may occur in regions such as the occipital cortex, which have high metabolic demand and neural activity, and this is consistent with previous reports of *OPA1*‐associated encephalopathies [8, 22]. This pattern may indicate that the occipital cortex, due to high neuronal activity and high metabolic demand, is more susceptible to damage in mitochondrial diseases and could serve as a site of seizure initiation.

Furthermore, this vulnerability may be amplified by systemic stressors. In our patient, the onset of SRSE occurred after nonspecific symptoms such as headache, fatigue, and vomiting, and the recurrence of seizures coincided with urinary tract infection and discontinuation of anticonvulsant medications. This suggests that infection and medication disruption can act as triggers for the metabolic decompensation and seizure in mitochondrial disorders.

### 4.3 Reported Genetic and Mitochondrial Contributors to SRSE

Despite extensive advances in imaging, infectious, and autoimmune investigations, the etiology of NORSE and SRSE remains unclear in many patients. Recent studies suggest that some of these cases may have underlying genetic or mitochondrial disorders, with status epilepticus as the first clinical manifestation. Behrndt et al. [40] in a systematic review studied 65 cases of NORSE who had variants in the *POLG* gene. In 75% of cases, refractory status epilepticus (RSE) occurred within 1 year of the first seizure, a severe clinical course that differs from classic mitochondrial epilepsy.

In addition, Ladds et al. [7] reported two children with a heterozygous variant in *DNM1L* (p.Arg403Cys) who developed super‐refractory status epilepticus after a mild viral illness or vaccination, with biochemical evidence of mitochondrial dysfunction. *OPA1* and *DNM1L* encode key regulators of mitochondrial dynamics and mediate opposing processes. *OPA1* promotes inner membrane fusion, whereas DRP1 (encoded by *DNM1L*) facilitates outer membrane fission. Disruption of either pathway can disrupt the integrity of the mitochondrial network, leading to neurodegeneration and epileptogenic vulnerability. Interestingly, both genes are implicated in refractory epilepsy syndromes, including mitochondrial SRSE, highlighting the delicate balance required between fusion and fission for neuronal homeostasis [41, 42, 43].

Furthermore Li et al. [9] reported a patient with compound heterozygous variants in the *TBC1D24* (c.1416_1437del; c.1499C>T) gene who presented with nonconvulsive status epilepticus (NCSE) and additional features such as ataxia and cerebellar atrophy, consistent with a broader neurogenetic phenotype.

A study has also investigated the role of *FASTKD2* in NORSE. A homozygous loss of function variant (c.1072C>T) in this gene has been reported in a 14‐year‐old male who initially presented with NORSE, followed by a clinical course of progressive focal epilepsy, optic atrophy, and unilateral cerebral atrophy. The *FASTKD2* gene plays a key role in mitochondrial RNA processing and mitochondrial ribosome biogenesis. Defects in the function of this gene can disrupt the synthesis of mitochondrial proteins and, as a result, disrupt cellular energy balance, a condition that may lead to increased neuronal sensitivity and the development of epilepsy in mitochondrial disorders [10].

Kellogg et al. [11] studied four patients with cryptogenic NORSE by gene panel testing: two patients had heterozygous pathogenic variants in the *POLG* (c.1399G>A) and PIGG (c.910C>T) genes, both of which are involved in energy metabolism, while the other two had multiple uncertain significance variants (VUS) in epilepsy‐related genes. These results raise the possibility that febrile or metabolic stress can activate latent defects in mitochondrial function and ultimately lead to the development of status epilepticus (SE) in individuals with a genetically predisposed background.

In a study, Giovannini et al. [12] identified a homozygous variant in the *FADD* gene in a family with recurrent cases of FIRES. The *FADD* gene encodes an important mediator protein in the apoptotic and immune signaling pathways, and its dysfunction can lead to impaired control of neuroinflammation, which could indicate a novel immunogenetic pathway in the pathogenesis of FIRES. Although FADD is not a mitochondrial protein, several studies show that it plays an important role in connecting death receptor signaling to mitochondrial dysfunction. Necroptosis, a programmed form of cell death triggered by inflammatory signals, depends on the RIP1–RIP3 complex, which drives excess mitochondrial ROS production, loss of ATP, and collapse of the mitochondrial membrane potential. FADD is a key mediator in this process [44]. Experimental work has also shown that FADD regulates T‐cell receptor–mediated necroptosis with downstream mitochondrial injury [45] and is necessary for TNF‐α–driven mitochondrial changes through caspase‐8 activation, independent of RIP and caspase‐3 [46]. Together, these findings suggest that FADD dysfunction can impair mitochondrial control and contribute to neuroinflammation, offering a plausible link to the pathogenesis of FIRES. Collectively, these findings emphasize the need for early genetic and metabolic testing in patients with cryptogenic SRSE, NORSE, or FIRES. Identification of a mitochondrial or genetic cause could prevent potentially harmful experimental immunosuppression and pave the way for more targeted therapeutic strategies.

### 4.4 Diagnostic Framework for Mitochondrial SRSE

Based on case reports and increasing genetic findings, a subset of SRSE patients has profound mitochondrial dysfunction in the absence of inflammatory, infectious, or autoimmune etiologies. These cases, including our study and cases with variants in *POLG, FASTKD2, DNM1L*, and *OPA1* genes, suggest that we may be facing a distinct clinical entity referred to as “Mitochondrial SRSE”. Mitochondrial SRSE should be considered as a metabolic decompensation syndrome resulting from a failure in cellular energy production in individuals with a genetically susceptible background, rather than as a subtype of autoimmune encephalitis. Early recognition of this clinical manifestation is essential for appropriate management of the disease.

Several clinical indicators can suggest the possibility of mitochondrial SRSE:

1. A history of neurological symptoms, such as optic atrophy, muscle weakness, developmental delay, or unexplained hypotonia in the period preceding the onset of seizures.
2. MRI abnormalities such as posterior‐predominant diffusion, especially in the occipital cortex, thalamus, or brainstem.
3. Absence of infectious or autoimmune evidence on paraclinical assessments, including unremarkable cerebrospinal fluid and serological findings.
4. Poor or paradoxical response to immunotherapy, in some cases accompanied by worsening of clinical presentations

In patients with suspected mitochondrial SRSE, empirical immunosuppressive therapy may not only be ineffective, but in some cases may worsen the course of the disease. For early diagnosis and effective management, a diagnostic algorithm is recommended:

1. In the initial presentation of SRSE, structural, infectious, and autoimmune causes must first be systematically ruled out through imaging, laboratory tests, and cerebrospinal fluid examination.
2. If initial evaluations are negative, further assessment should include: (A) a detailed review of personal and family history for evidence of neuromuscular or visual disorders, (B) brain imaging findings suggestive of metabolic involvement, particularly abnormal diffusion patterns in posterior brain regions, (C) clinical findings such as lactic acidosis or encephalopathy of unclear etiology should be considered.
3. Genetic testing, particularly WES, is recommended. Immunosuppressive therapy should only be considered if there is clear evidence of active inflammation.
4. Metabolic supportive therapy is often considered, including administration of mitochondrial cocktails (such as coenzyme Q10, L‐carnitine, riboflavin), although robust evidence for their efficacy is still lacking. In contrast, avoidance of mitochondrial toxins such as valproate and propofol is better supported by clinical reports of severe adverse outcomes in mitochondrial disease [47].

This proposed algorithm provides clinicians with a practical and systematic approach to distinguishing between mitochondrial SRSE and autoimmune or cryptogenic variants. Although further studies are needed to formally confirm this subgroup, mounting clinical and genetic evidence strongly supports the recognition of mitochondrial SRSE as a distinct diagnostic category within the overall SRSE framework.

### 4.5 Management of SRSE Depends on Its Etiology

Given the growing understanding of the role of genetic and mitochondrial factors in SRSE, it is essential to review existing therapeutic methods based on empirical approaches. Empiric treatment often involves early use of aggressive immunotherapies such as high‐dose corticosteroids, IVIG, and second‐line agents like rituximab. These approaches are based on the assumption of autoimmune or parainfectious causes, especially in cryptogenic cases. However, in patients with mitochondrial dysfunction or other genetic abnormalities, these approaches can be detrimental, as they may increase metabolic stress, worsen the course of the disease, delay the correct diagnosis, and/or precipitate opportunistic infection [48].

Some mitochondrial disorders, including those with variants in the *POLG, FASTKD2*, and *OPA1* genes, may have severe neurological manifestations such as SRSE. In these patients, caution is warranted with certain immunotherapies. The use of corticosteroids may disrupt metabolic balance, as glucocorticoids have been shown to increase oxidative protein damage and impair mitochondrial function [47, 49, 50]. Similarly, IVIG has recognized risks of renal dysfunction, thromboembolic events, and cardiovascular complications, while plasmapheresis can also induce hemodynamic instability [51, 52]. In addition, cytotoxic drugs such as cyclophosphamide carry the risk of severe cardiotoxicity and, in rare cases, multiorgan failure [53, 54, 55].

These findings highlight the importance of early genetic and metabolic evaluation. Although empiric treatment is often critical in the early stages of presentation, the continuation of aggressive therapy should be reconsidered if infectious and autoimmune tests are negative. Our case illustrates the clinical importance of this issue, as the identified variants in *OPA1* were not fully considered in acute treatment decisions before the patient was transferred to MGH. This suggests that ignoring or delaying the use of genetic background in the early stages of treatment can lead to ineffective or even harmful treatment decisions.

In *OPA1*‐associated mitochondrial disorders, early diagnosis allows for a shift in treatment from invasive interventions to supportive care, such as the use of coenzyme Q10 (though evidence for its efficacy remains limited), a ketogenic diet, and avoidance of valproate or propofol. Propofol has been reported to impair mitochondrial function and, in susceptible individuals, to unmask previously subclinical mitochondrial disease, particularly in the setting of high‐dose or prolonged infusions [56]. On this basis, propofol‐related toxicity, including propofol infusion syndrome (PRIS), was carefully considered in our patient. However, there was no clinical or biochemical indication that propofol contributed to the presentation or subsequent course: metabolic acidosis, arrhythmia, heart failure, renal or hepatic dysfunction, rhabdomyolysis, or other features suggestive of PRIS were not observed. Ultimately, integrating genomic testing into early SRSE evaluation helps distinguish inflammatory from metabolic etiologies and open pathways to more precise, less harmful management strategies.

Identifying the common endophenotype associated with epileptic manifestations of mitochondrial SRSE is critical to developing novel therapies. Until more is known, our understanding of mitochondrial SRSE may need to be extrapolated from better‐studied mitochondrial disorders associated with epilepsy. For example, in *POLG*‐related disease, epilepsy is a common presenting feature, occurring in 50%–65% of adults and up to 80% of pediatric cases [57]. The quantitative loss of mtDNA in neurons is considered the earliest and most important upstream event in the pathophysiological cascade, leading to loss of mitochondrial respiratory chain components and a progressive deficiency in energy metabolism [57]. Notably, seizure activity in these cases often precedes the appearance of cortical focal lesions, which are thought to result from cortical necrosis in the context of energy imbalance [57].

**FIGURE 3:**
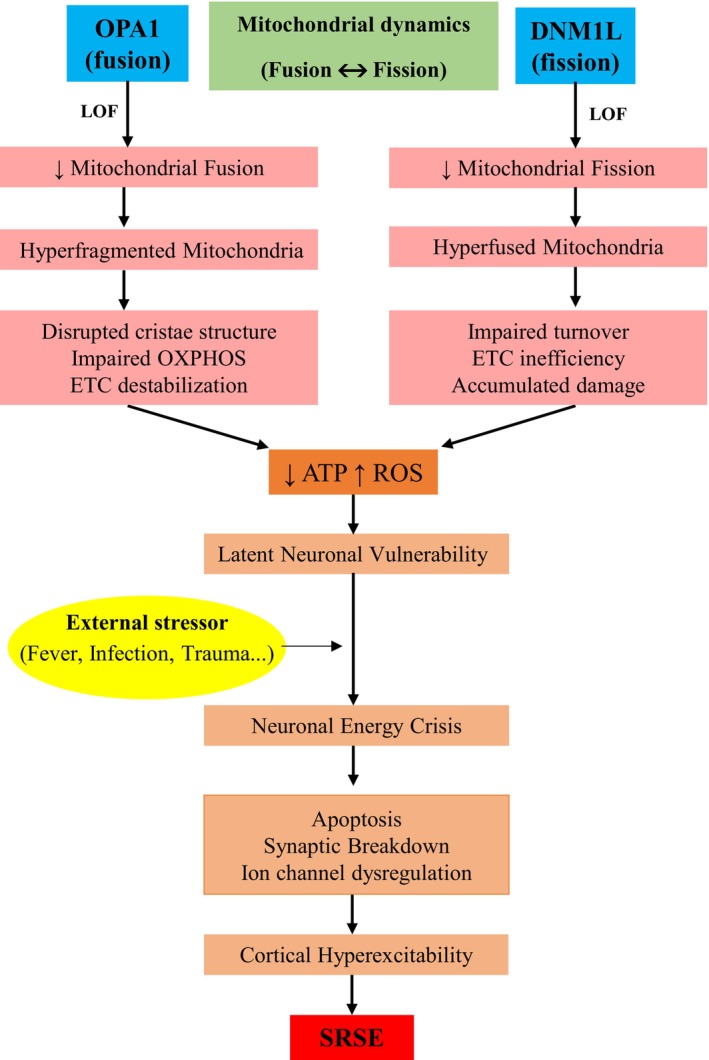
Proposed Pathophysiological pathway from Mitochondrial Fusion/Fission Defects to NORSE Schematic representation comparing biallelic loss of function (LOF) variants in *OPA1*, which impair mitochondrial fusion and produce a hyper fragmented (‘pro‐fission’) mitochondrial phenotype, with LOF variants in *DNM1L*, which impair fission and result in hyperfused mitochondria. Both phenotypes disrupt cristae structure, oxidative phosphorylation (OXPHOS), and electron transport chain (ETC) stability or efficiency, leading to reduced ATP production and increased reactive oxygen species (ROS). This latent neuronal vulnerability, when combined with an external stressor (e.g., fever, infection, trauma), can precipitate a neuronal energy crisis, triggering apoptosis, synaptic breakdown, and ion channel dysregulation. The resulting cortical hyperexcitability can culminate in Super Refractory Status Epilepticus (SRSE).

By contrast, variants in *DNM1L* and *OPA1* are not commonly associated with epilepsy or status epilepticus at high prevalence. These variants exert opposite effects on mitochondrial morphology (Figure 3), yet they converge on common downstream abnormalities in mitochondrial function, including OXPHOS defects, loss of cristae integrity, reduced ATP production, and increased ROS generation [58, 59, 60]. The loss of mtDNA in *OPA1*‐related disorders may also correlate with disease severity, since mtDNA deletion is not observed in milder *OPA1*‐related conditions such as ADOA Kjer type or ADOAD, but has been associated with more severe *OPA1*‐related presentations including ADOA plus [61]. In our case series, epileptic presentations of *OPA1*‐related disease also appeared to be linked to more severe compound loss‐of‐function variants in *OPA1*, such as missense LOF in combination with frameshift. Experimental evidence further supports this, as amelioration of *OPA1*‐related hyper fragmentation in mouse embryonic fibroblasts (MEFs) (e.g., via genetic modification, pgs1 deletion) [62] was shown to rescue abnormal mitochondrial respiration but no other abnormalities including cristae dysfunction, apoptotic sensitivity, or mtDNA depletion. This observation suggests that additional functions of *OPA1* beyond mitochondrial morphology are implicated in the pathophysiology of its related conditions. Taken together, these findings raise the possibility that loss of mtDNA may be involved in the development of *OPA1*‐related SRSE. Although mtDNA deletion itself may not be reversible, therapies aimed at ameliorating its downstream consequences or preventing its development could represent a high‐yield approach in this setting.

In terms of treatment strategies, if there is any hope of addressing acute manifestations of *OPA1*‐related and other mitochondrial disorders, the most accessible options may come from therapies already applied in the chronic setting. Unfortunately, even these remain limited. The ubiquinone analog coenzyme Q10 (CoQ10), though widely used, has not demonstrated efficacy for optic atrophy associated with *OPA1* [63]. More encouragingly, chronic treatment with idebenone for 7 weeks has shown improvements in visual acuity in patients with DOA [63]. In addition, tolfenamic acid, an FDA‐approved COX inhibitor, has recently been identified through a repurposing screen as capable of suppressing mgm1/opa1 phenotypes in yeast, with subsequent validation in MEFs and patient fibroblasts [64], and may represent a suitable candidate for clinical trials. However, it remains unresolved whether such interventions will also prove effective for seizure‐related phenotypes of *OPA1*‐related disorders. Collectively, these observations suggest that identifying the most efficacious treatments for mitochondrial SRSE will require not only a broader therapeutic arsenal but also a deeper understanding of the disorder itself.

## 5 Conclusion

The case presented in this study, together with recent studies, suggests that mitochondrial SRSE should be recognized as a distinct clinical subtype, distinct from autoimmune or cryptogenic forms, and should be investigated and treated differently. Because this type of SRSE results from genetic defects in mitochondrial function and cellular energy metabolism, initiating immunosuppressive therapies without an accurate diagnosis can be harmful. Therefore, early diagnosis with genetic testing can prevent potential iatrogenic harm and facilitate more appropriate therapeutic approaches. To improve therapeutic and diagnostic strategies in patients with genetic or mitochondrial SRSE, a number of key strategic measures should be prioritized. One of the most important is the creation of an international database that collects information on patients with or suspected of having a genetic or mitochondrial SRSE. Such a registry would allow for more accurate prevalence monitoring, enhance genotype–phenotype analyses, and facilitate the identification and validation of potential biomarkers. Clinical guidelines should be revised to increase diagnostic accuracy in SRSE patients. Mitochondrial screening should be included in the initial evaluation, especially in cases with early optic atrophy, metabolic stroke patterns on imaging, or poor response to immunotherapy.

From a therapeutic perspective, current evidence emphasizes the urgent need for targeted and large‐scale studies of mitochondrial function‐based interventions in patients with status epilepticus. Supplements such as coenzyme Q10, carnitine, riboflavin, and a ketogenic diet are effective in enhancing metabolic resistance. Also, compounds with potential neuroprotective effects such as ganaxolone and KH176 are promising.

Functional studies using neuronal models derived from induced pluripotent stem cells (iPSCs) can show how specific variants, such as p.Ile382Met in the *OPA1* gene, cause neural network hyperexcitability and energy depletion under stress conditions. These platforms can also facilitate the screening process for personalized medicine and support the development of mechanism‐based pharmacological interventions.

Overall, this study demonstrates the link between molecular pathology and clinical decision‐making in the management of SRSE. Our findings show how understanding the mechanisms of mitochondrial disorders, particularly those involving disease‐causing variants in the *OPA1* gene, can provide more precise, personalized, and ultimately safer therapeutic strategies for SRSE patients. Mitochondrial SRSE is a major challenge in the advancement of precision medicine, but it also presents a valuable opportunity to advance therapeutic approaches. Timely diagnosis, application of appropriate therapeutic strategies, and development of a global research platform are crucial to improve prognosis and reduce the clinical burden of this neurologic emergency.

## Author Contributions

P.M.: investigation, writing – initial draft, writing – review and editing. L.B.: data curation, investigation. C.M.M.: conceptualization, data curation, investigation, supervision, writing – review and editing.

## Funding

CMM was supported by NIH/NINDS K08NS118107.

## Ethics Statement

All procedures in studies involving human participants were performed in accordance with the ethical standards of the institutional ethics committee of Northwestern University and with the 1975 Helsinki Declaration and its later amendments or comparable ethical standards. Informed consent was obtained from all participants included in the study.

## Conflicts of Interest

The authors declare no conflicts of interest.

## Data Availability

The data that support the findings of this study are available on request from the corresponding author. The data are not publicly available due to privacy or ethical restrictions.
